# When the cure turns cold: ischaemic appendicitis as a rare complication of ileocolic embolization

**DOI:** 10.1093/jscr/rjag117

**Published:** 2026-03-11

**Authors:** Mashaal Hamayun, Mina Sarofim, Andrew Gilmore

**Affiliations:** Department of Colorectal Surgery, Liverpool Hospital, Corner of Elizabeth and Goulburn Streets, Liverpool, NSW 2170, Australia; Liverpool Hospital, Innovation, Surgical Teaching and Research Unit, Corner of Elizabeth and Goulburn Streets, Liverpool, NSW 2170, Australia; Department of Colorectal Surgery, Liverpool Hospital, Corner of Elizabeth and Goulburn Streets, Liverpool, NSW 2170, Australia; Liverpool Hospital, Innovation, Surgical Teaching and Research Unit, Corner of Elizabeth and Goulburn Streets, Liverpool, NSW 2170, Australia; Faculty of Medicine and Health, University of Sydney, Parramatta Road, Sydney, NSW 2006, Australia; Department of Colorectal Surgery, Liverpool Hospital, Corner of Elizabeth and Goulburn Streets, Liverpool, NSW 2170, Australia; Liverpool Hospital, Innovation, Surgical Teaching and Research Unit, Corner of Elizabeth and Goulburn Streets, Liverpool, NSW 2170, Australia; School of Medicine, Western Sydney University, Kingswood Avenue, Penrith, NSW 2751, Australia; Faculty of Medicine and Health Sciences, Macquarie University Hospital, Technology Place, Macquarie Park, NSW 2109, Australia

**Keywords:** appendicitis, angioembolization, interventional radiology, gastrointestinal bleeding

## Abstract

In many tertiary centers with rapid access and expertise, IR angioembolization is now frequently employed as first line treatment for control of active and potentially life-threatening hemorrhage, rather than endoscopic or surgical management. We present an extremely rare case of acute perforated appendicitis following ileocolic angioembolization, for the management of major colonic bleeding. Although acute appendicitis is a very common general surgical pathology, its occurrence secondary to angioembolization, representing an ischemic complication, has only been described once before in the literature. It is thus important to maintain close clinical observation for signs of ischemia following gastrointestinal embolization procedures, particularly when performed in the ileocolic region due to its limited collateral blood supply. While non-operative management was successfully pursued, as more cases are published in the literature, a consensus guideline can ideally be developed to determine the optimal management for this rare subset of patients.

## Introduction

We present a rare case of acute perforated appendicitis following ileocolic angioembolization, for the management of major colonic bleeding. Although acute appendicitis is a very common general surgical pathology [1], this is the second recorded case in the literature [2] to occur secondary to angioembolization.

## Case report

**Figure 1:**
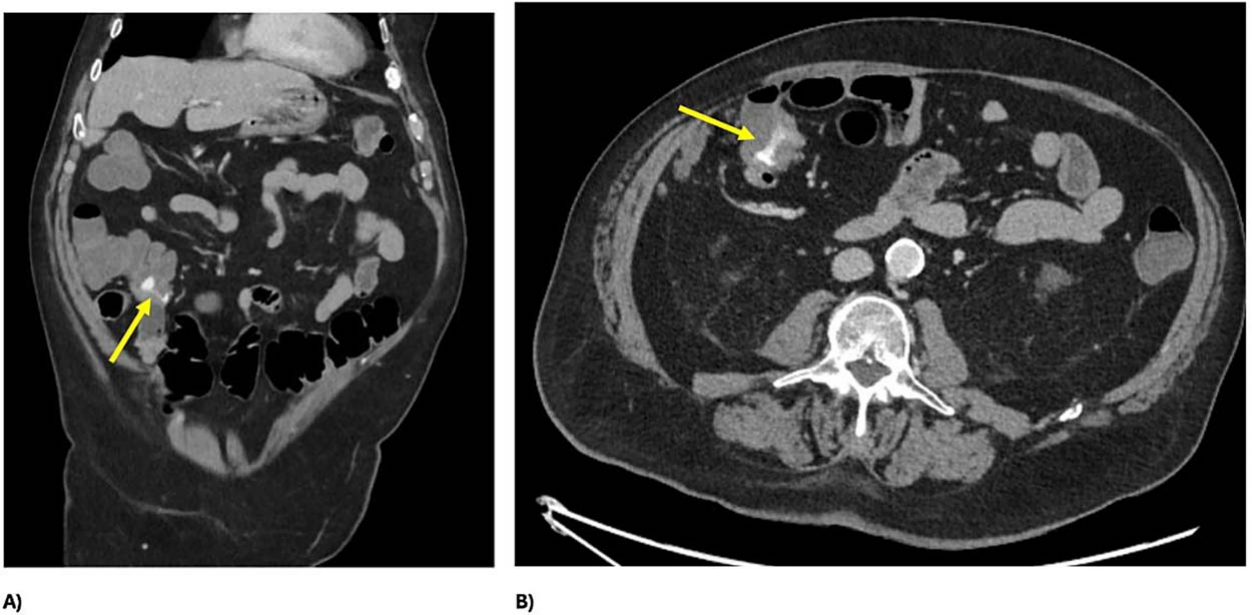
CT demonstrating active bleeding pre-embolization, with pooling of contrast in the caecum and appendiceal base; (A) axial view and (B) coronal view.

**Figure 2:**
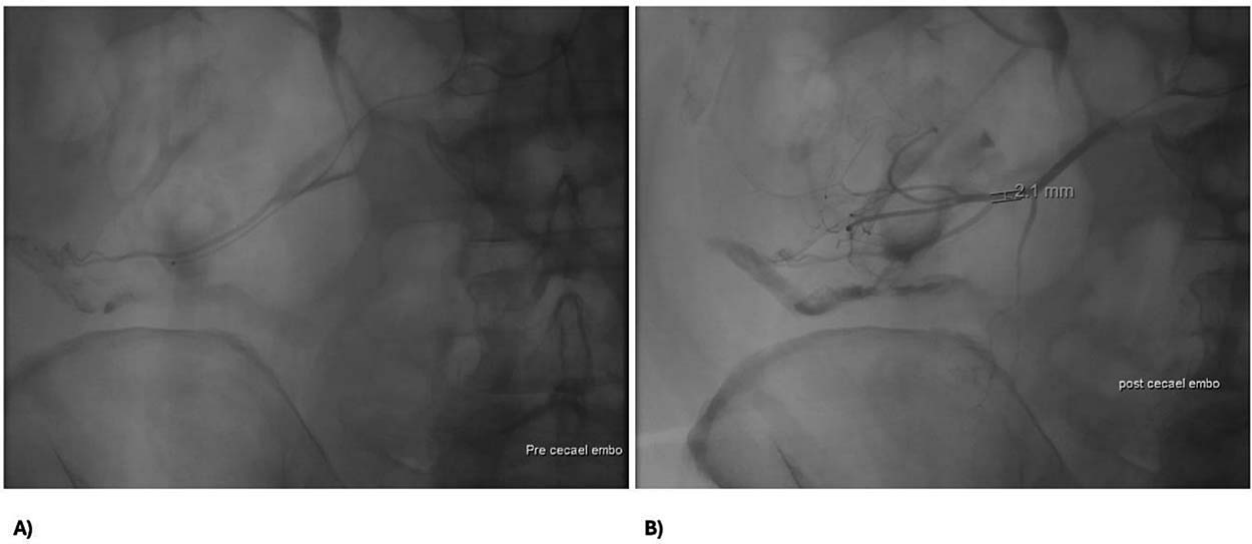
Abdomen angiography demonstrating successful ileocaecal angioembolization with an embolic gel.

A 73 year-old male presented to our tertiary hospital with a three day history of major colonic bleeding, while on therapeutic warfarin for a metallic aortic valve. Other medical history included laparoscopic cholecystectomy, Parkinson’s disease, and a normal colonoscopy 12 months prior. Computed tomographic mesenteric angiography demonstrated active contrast pooling within the caecum and appendiceal base (Fig. 1). He underwent successful interventional radiology guided angioembolization of a branch of the ileocolic artery, using a gel embolic injection (Fig. 2).

**Figure 3:**
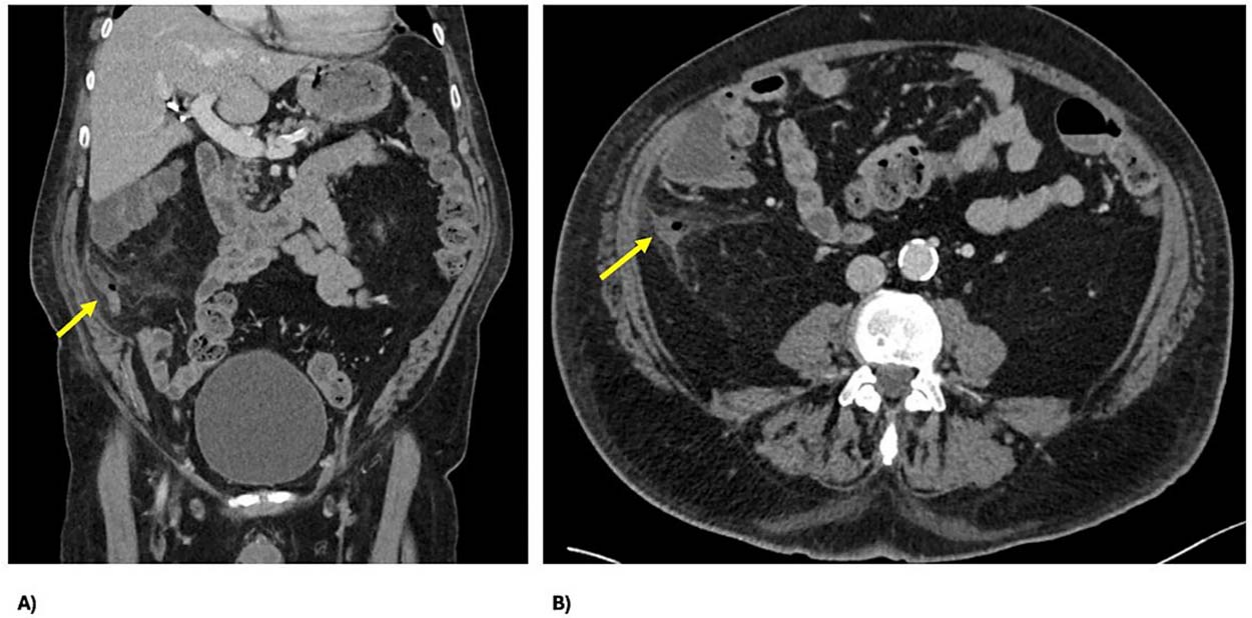
CT demonstrating perforated appendicitis post-embolization with a fluid collection and stranding, with the line of demarcation demonstrating hypoperfusion, suggestive of ischemia; (A) axial view and (B) coronal view.

Two days post-embolization, the patient developed right-sided abdominal pain, localized abdominal tenderness and elevated inflammatory markers (white cell count 17 × 10^9^/L, C-reactive protein 42 mg/L). Repeat computed tomography (CT) abdomen/pelvis showed perforated appendicitis with appendiceal mural thickening and extraluminal gas, not present on his initial imaging (Fig. 3). Treated non-operatively with intravenous antibiotics, his signs and symptoms resolved within 24 hours. He continued his recovery and was subsequently discharged on Day 11 for consideration of interval appendicectomy.

## Discussion

Interventional radiology (IR) has transformed many areas of modern medicine, including the management of acute lower gastrointestinal (GI) bleeding. In many tertiary centers with rapid access and expertise, IR angioembolization is now frequently employed as first line treatment for control of active and potentially life-threatening hemorrhage, rather than endoscopic or surgical management. It offers an enhanced ability to identify and target intraluminal bleeding sites, is minimally invasive with shorter recovery time, shortens length of hospital stay and reduces morbidity and mortality profile [3]. IR angioembolization selectively occludes (or significantly decreases blood flow) to specific bleeding vessels, while still preserving collateral perfusion of the small or large bowel [4]. Embolization may be achieved via temporary (gelatin) or permanent measures (coils, glue, polyvinyl alcohol) [4].

While innovative, angiographic embolization is technically challenging and has potential complications, which can be categorized by puncture site, embolization site and post-embolization adverse outcomes. A systematic review reported a complication rate of 5%–9% for IR angiographic embolization of GI bleeding [5]. At the puncture site there can be pain, bleeding, hematoma, infection or rarely a pseudoaneurysm [4]. At the embolization site (the most common) is the risk of bowel ischemia and infarction [4]. Gelatin embolic, which was used in our patient, is associated with a higher risk of ischemia due to its distal embolization [4]. Post-embolization complications include systemic inflammatory response, rebleeding secondary to collaterals or coil migration, colonic ischemia and delayed stricture [4].

This is the second case report in the literature to describe appendicitis following angioembolization of the ileocolic vessel, representing an arterial ischemic complication successfully managed non-operatively with intravenous antibiotics. It reflects the need for careful patient and target selection, and close monitoring post-embolization, as we learn more about potential complications.

The appendix, once considered a vestigial organ with an unclear function, is now recognized as an important part of the immune system. Appendicitis has a lifetime incidence of 7%–8%, with a slightly greater preponderance in men [1]. Typical symptoms are migratory right iliac fossa pain, nausea, vomiting, fevers and anorexia [1]. Multiple theories exist as to the etiology of appendicitis including diet, trauma, genetics, infection (e.g. viruses, parasites, tuberculosis), along with obstructive causes such as faecoliths, lymphatic hyperplasia and neoplasia (e.g. neuroendocrine tumors, mucinous tumor, and adenocarcinoma) [1].

The pathophysiology of appendicitis typically involves increased intraluminal and intramural pressure causing venous ischemia, followed by large vessel arterial ischemia, and subsequently gangrene, with bacterial overgrowth of *Escheria coli*, Bacteriodes, or Pseudomonas [1]. Untreated, this may be complicated by perforation, usually at the 48 hour mark. This risk of perforation increases by 5% at every 12 hours after that [1]. In elderly patients, perforation is highly associated with ischemia [1]. In our patient’s case, while the coincidence of acute appendicitis cannot be excluded, it is unlikely based on the sequence of events. Instead, interestingly, this is likely an arterial ischemic pathology exacerbated by the limited collateral blood supply in the ileocolic region.

The treatment pathway for appendicitis diverges into non-operative or operative management. The gold standard in Western countries continues to be laparoscopic appendicectomy [6]. It carries a 90% complication-free treatment success rate versus 68% for non-operative management [6]. Many studies now show that in select patients (those with early, uncomplicated appendicitis and no faecolith) non-operative management with antibiotics is safe and successful [6]. These patients are to be cautioned regarding the 20–30% risk of recurrence within 5 years, and those over 40 years may require an outpatient colonoscopy and interval appendicectomy [6]. Our patient was therapeutically anticoagulated for a metallic valve, plus symptoms rapidly improved and thus non-operative management was successfully pursued. As more cases are published in the literature, a consensus guideline can ideally be developed to determine the optimal management for this rare subset of patients.

In conclusion, this is a highly unusual and rare incidence of acute appendicitis due to ischaemia from IR guided ileocolic angioembolization, raising awareness of potential complications. Close clinical observation following GI embolization procedures thus remains important. The choice to recommend operative, or non-operative, management of appendicitis is made on a case by case basis.
